# Exception from Informed Consent and Waiver of Informed Consent for Comparative Effectiveness Trials in Emergency Care Research

**DOI:** 10.1002/eahr.70001

**Published:** 2025-12-31

**Authors:** Jonathan D. Casey, Matthew W. Semler, Jeremy Brown, Clifton W. Callaway, Stephanie C. DeMasi, Neal W. Dickert, Luke Gelinas, Francis X. Guyette, Ann R. Johnson, Wesley H. Self, Todd W. Rice, Stephanie R. Morain

**Affiliations:** ^1^ Assistant professor of medicine in the Division of Allergy, Pulmonary, and Critical Care Medicine at the Vanderbilt Center for Learning Healthcare and the Vanderbilt Institute for Clinical and Translational Research at Vanderbilt University Medical Center; ^2^ Associate professor of medicine in the Division of Allergy, Pulmonary, and Critical Care Medicine at the Vanderbilt Center for Learning Healthcar, and the Vanderbilt Institute for Clinical and Translational Research at Vanderbilt University Medical Center; ^3^ Director of the Office of Emergency Care Research at the National Institute of Neurological Disorders and Stroke; ^4^ Professor in the Department of Emergency Medicine at the University of Pittsburgh; ^5^ Assistant professor in the Department of Emergency Medicine at Vanderbilt University Medical Center; ^6^ Professor in the Division of Cardiology in the Department of Medicine and the Emory Health Services Research Center at Emory University School of Medicine; ^7^ Senior IRB chair director at Advarra; ^8^ Professor of emergency medicine at the University of Pittsburgh; ^9^ Associate vice provost in the Research Compliance Office at Stanford University; ^10^ Professor of emergency medicine in the Vanderbilt Institute for Clinical and Translational Research and the Department of Emergency Medicine at Vanderbilt University Medical Center; ^11^ Professor of medicine in the Division of Allergy, Pulmonary, and Critical Care Medicine at the Vanderbilt Center for Learning Healthcare and the Vanderbilt Institute for Clinical and Translational Research at Vanderbilt University Medical Center; ^12^ Associate professor at the Berman Institute of Bioethics and in the Department of Health Policy and Management at Bloomberg School of Public Health

**Keywords:** human subjects research, human research ethics, emergency medicine research, informed consent, institutional review boards, human research regulations

## Abstract

For many of the decisions made in the clinical care setting, clinicians lack evidence to inform which treatment would result in the best patient outcomes. This problem is particularly common in emergency care, a field in which the condition of the patient and the urgent nature of the treatment often preclude research conducted using prospective informed consent. Large‐scale comparative effectiveness clinical trials could address the evidence gaps in clinical medicine and improve patient outcomes but are hindered by the lack of a clear regulatory framework in the United States for low‐to‐minimal risk trials comparing commonly used treatments. In this paper, we summarize a presentation and discussion that took place at a workshop held by the National Institutes of Health that focused on the issue of informed consent and the appropriate regulatory pathway for comparative effectiveness trials conducted in the emergency care setting. A key insight of this workshop is that generating the comparative effectiveness data needed to improve clinical care will require revising ethical and regulatory oversight practices and related guidance to support the conduct of this socially valuable research.

In daily clinical practice, clinicians must often decide between two or more commonly used treatments for which little or no evidence is available to inform which treatment will produce the best patient outcomes. The absence of high‐quality evidence causes variability in the care patients receive that is unrelated to knowledge of what is best for the patient and instead derives from non‐evidentiary factors like the clinician's subspecialty, the practice patterns of the local hospital, or pharmaceutical industry marketing. As a result, an individual patient commonly would receive completely different treatments based on where, when, and from whom she or he receives care. This arbitrary variation in clinical care systematically exposes patients to treatments that are ineffective or even harmful.^1^

Emergency tracheal intubation is an example of a procedure for which a lack of evidence has led to dramatic variation in the tools, techniques, and medications that patients are exposed to in everyday clinical care. Millions of critically ill patients undergo tracheal intubation in an emergency department or intensive care unit each year. Unlike elective intubation in an operating room during which complications are uncommon, as many as 40% of patients undergoing tracheal intubation in an emergency department or intensive care unit experience hypoxemia or hypotension. Between 1% and 3% of patients experience cardiac arrest or death. Few other circumstances in medicine are so serious that patients experience a 1‐in‐50 chance of dying in the next two minutes. Yet strikingly little evidence has been available to help clinicians make the dozens of treatment decisions that must be made before, during, and immediately after every tracheal intubation procedure. Despite millions of critically ill adults undergoing tracheal intubation each year in clinical care, not a single multicenter randomized clinical trial comparing approaches to emergency tracheal intubation was conducted in the United States in the 40 years after the procedure was introduced to practice (in 1970).^2^

A primary cause of this evidence gap is the lack of a regulatory paradigm for clinical trials comparing the effectiveness of common treatments that patients are already receiving as part of clinical care. Written informed consent to participate in research is not feasible for many comparative effectiveness questions, particularly in the emergency care setting. During emergency tracheal intubation, for example, most patients are comatose or delirious and surrogate decision‐makers are commonly unavailable. Even when a patient does have the capacity to provide informed consent or a surrogate is immediately available, the time between recognition of the need for tracheal intubation and initiation of the procedure (often five minutes or less) and the clinical status of the patient (experiencing a life‐threatening illness requiring numerous simultaneous interventions) frequently preclude execution of a meaningful informed consent process. Attempts to obtain informed consent from the patient or permission from a surrogate in these circumstances would delay emergency care and worse yet, could be perceived by patients or surrogates as coercive.

When conducting research and obtaining written informed consent is impracticable, two mechanisms for conducting such research exist in the US: (1) exception from informed consent (EFIC) requirements for emergency research^3^ and (2) exception from informed consent requirements for minimal risk clinical investigations (referred to here as “waiver of informed consent”).^4^ How and when each mechanism should be used for comparative effectiveness trials in the emergency care setting remains unclear, and was the focus of a one‐day, in‐person workshop held by the National Institutes of Health (NIH) entitled “Regulatory Determinations Related to Consent, EFIC, and Waiver of Consent in Emergency Clinical Trials.” Held on March 12, 2024, the workshop included emergency care researchers, institutional review board (IRB) leaders, funders, and members of the US Food and Drug Administration (FDA) and the Office for Human Research Protections (OHRP). The workshop included five sessions, each of which started with a case study, followed by an open discussion among all workshop attendees. For the session summarized in this manuscript, “Drug Choices for Rapid Sequence Intubation and Comparative Effectiveness,” a member of the Pragmatic Critical Care Research Group^5^ was invited to present examples of clinical trials conducted using both EFIC and a waiver of informed consent and to discuss the regulations relevant to each. The following sections summarize the presentation from the Pragmatic Critical Care Research Group member and the discussion that followed.

## OVERVIEW OF REGULATORY GUIDELINES

In 1996, the FDA established the EFIC regulations to create a regulatory approach to emergency care research when it is not feasible to obtain a patient's informed consent to participate in a study. To qualify for EFIC, a study must, among other criteria, focus on a life‐threatening emergency condition for which currently available treatments are unproven or unsatisfactory and evaluate an intervention that must be delivered within a time window that is too short to obtain a patient's informed consent to participate in the study.^3^ The EFIC regulations are well‐suited to trials that evaluate new drugs, devices, or biologics, and involve greater than minimal risk because the regulations require: (1) the prospect of direct benefit to participants; (2) that the risks of the trial must be reasonable in relation to the risks and benefits of the condition and current therapy; and (3) that investigators consult with the communities in which the research will occur and from which the participants will be drawn during the design of the trial. Investigators must complete a campaign of public disclosure and community consultation to inform community members of the plan to conduct the research and solicit concerns from the community prior to beginning the study. Study teams must provide an opportunity for patients, legally authorized representatives, or family members to decline participation whenever feasible, and must notify patients, legally authorized representatives, or family members of enrollment in the research at the earliest feasible opportunity to provide an option to discontinue participation. Investigators are also required to notify the community of the results of the research at the end of the study. The FDA also requires that all trials involving a drug, device, or biologic conducted under EFIC receive FDA oversight through an Investigational New Drug (IND) application or Investigational Device Exemption (IDE), even if the interventions are commercially available and being used within their approved indications. Additional requirements are listed in the relevant regulations.^3^

The EFIC regulations, which were also adopted by the OHRP, have provided a clear pathway to conduct research in important areas such as cardiac arrest,^6^ hemorrhagic shock,^7^ traumatic brain injury,^8^ status epilepticus,^9^ and ischemic stroke.^10^ However, many emergency conditions, including cardiogenic shock, mechanical ventilation, and acute agitation are either considered to be non‐life‐threatening or are treated with interventions that have insufficiently short intervention windows to fit the regulatory criteria and cannot be conducted using EFIC. In addition, the requirements of EFIC are complex, and meeting them is costly and time‐consuming. The pretrial EFIC processes of community consultation and public disclosure have been reported to require up to 3 years and $50,000 per clinical trial site.^11^

More importantly, EFIC trials require significant research infrastructure to meet requirements that patients, legally authorized representatives, or families (1) be provided with an opportunity whenever feasible to decline participation before enrollment and (2) be notified of enrollment at the earliest feasible opportunity after enrollment and be provided an opportunity to discontinue participation. To meet these requirements, investigators must either ensure around‐the‐clock presence of research staff or limit enrollment to sites and periods in which research staff are immediately available. Experts state that the complexity of the EFIC regulations and the cost and time required to comply with them have slowed the conduct of randomized trials in the emergency care setting.^11^ For example, in the first 20 years following the release of EFIC regulations, only 41 randomized trials using EFIC were registered with the FDA (approximately two trials per year).^12^ In comparison, over a similar time period, 42 critical care trials examined the use of corticosteroids in sepsis (a single intervention for a single condition)—more than all EFIC trials combined.^13^ With regard to emergency tracheal intubation (the example procedure described above), no multicenter randomized trial comparing different approaches to emergency tracheal intubation using EFIC has ever been completed.

Waiver of informed consent for minimal risk research is an alternative pathway to conducting research when obtaining informed consent from research participants is not practicable. Waiver of informed consent

**We believe that the inability to efficiently conduct comparative effectiveness trials leads to arbitrary variation in clinical care that systematically exposes patients to suboptimal or harmful therapies.**

for minimal risk research has long been permitted by the OHRP under conditions set forth in the Common Rule—conditions that have recently been adopted by the FDA.^14^, ^15^ To qualify for a waiver (or alteration) of informed consent under this distinct regulatory pathway, a study must: (1) represent no more than minimal risk; (2) be impracticable to conduct without a waiver or alteration of informed consent; (3) only use identifiable private health information if such information is required to conduct the study; (4) be such that waiving consent does not adversely affect research participants’ rights or welfare; and (5) whenever appropriate, include provisions to provide research participants with pertinent information after participation in the study. For research in which obtaining informed consent for participation is impracticable, the determination of whether the research may be conducted under these criteria for waiver of consent depends largely on the assessment of whether participating in the research represents minimal risk, which has engendered controversy in the research community. Some bioethicists have reasoned that when two treatments are commonly used in clinical care and neither is known to be superior, and when a clinician thinks either treatment would be a reasonable choice for the patient, having that choice made by randomization as part of a clinical trial rather than by arbitrary factors unrelated to knowledge of best therapy for a specific patient (e.g., the specialty of the clinician, the practice patterns of the hospital), may represent no more than minimal incremental risk, and would thus potentially qualify for a waiver or alteration of consent.^16^, ^17^, ^18^, ^19^, ^20^, ^21^

Others object to this assessment. For example, in draft guidance released in 2014,^22^ the OHRP asserted that the risks of the standards of care being evaluated as a purpose of the research should be considered risks of research, which could be interpreted as precluding studies comparing any two treatments a patient would receive in clinical care under a waiver of informed consent. This position is arguably in conflict, however, with guidance in the Common Rule, which states, “In evaluating risks and benefits, the IRB should consider only those risks and benefits that may result from the research (as distinguished from risks and benefits of therapies subjects would receive even if not participating in the research).”^23^ The 2014 OHRP guidance has not been finalized, but remains publicly available in a draft version, creating regulatory ambiguity for IRBs and for investigators seeking to address critical evidence gaps about the treatments patients are receiving daily in clinical care.

## CURRENT GUIDANCE ON REGULATORY PATHWAY FOR COMPARATIVE EFFECTIVENESS RESEARCH

In a recent article, Robert Califf, who was the FDA commissioner at that time, stated that comparative effectiveness trials of therapies used in clinical care “offer the potential to generate information to improve clinical practice and public health policy, and, in turn, to enable major improvements in the health status of Americans” and that many of these trials would “pose very little, if any, additional risk compared to ongoing care.” Califf noted, however, that “Neither HHS nor FDA regulations currently have guidance on whether or when studies of this sort might be categorized as minimal risk …These issues need the joint attention of federal agencies, the research community, the health care delivery ecosystem, and patient advocates.”^24^

In the absence of clear federal guidance, minimal risk determinations for trials comparing the effectiveness of treatments used in clinical care have been left to IRBs.^25^ Some IRBs have interpreted the 2014 OHRP draft guidance as meaning that any trial that uses randomization is inherently greater than minimal risk and cannot be conducted under a waiver of informed consent. Other IRBs have approved, with a waiver of informed consent, randomized trials comparing commonly available treatments that patients would receive as a part of standard clinical care.^26^ Recent examples of comparative effectiveness trials that enrolled patients under a waiver of informed consent include trials from a broad range of clinical domains, including tracheal intubation,^27^ mechanical ventilation,^28^ stroke,^29^ cardiac arrest,^30^ infection prevention,^31^ and population health.^32^ In 2023 alone, randomized trials conducted under a waiver of informed consent enrolled more than one million patients in the US^31^, ^32^ compared to fewer than 50,000 patients enrolled in all of the trials conducted in the first 20 years of EFIC combined.^12^

## EXAMPLES OF RECENT CLINICAL TRIALS UNDER EFIC VERSUS WAIVER OF INFORMED CONSENT

### The PREOXI trial

The Pragmatic Trial Examining Oxygenation Prior to Intubation (PREOXI) trial compared preoxygenation with noninvasive ventilation versus preoxygenation with an oxygen mask during emergency tracheal intubation of critically ill adults. Both noninvasive ventilation and an oxygen mask are approved devices received by millions of patients undergoing tracheal intubation in the emergency department or intensive care unit each year in the US. Before the PREOXI trial, evidence to inform the choice between the two devices was limited and neither was known to be superior. The trial was funded by the US Department of Defense with a total budget of approximately $1.5 million. The trial protocol was completed in September 2021 and was approved by the IRB at Vanderbilt University Medical Center with a waiver of informed consent with secondary concurrence by the Defense Health Agency Office of Research Protections of the Department of Defense. The first patient‐participant was enrolled in March 2022, less than six months after completion of the trial protocol. Following enrollment, clinicians provided participants, their legally authorized representatives, or their families with an IRB‐approved document that described participation in the trial, the rationale for the trial, the two treatment approaches being compared, their risks and benefits, and other aspects of the research. The document also provided the contact information for study personnel and an opportunity to discontinue participation.

**Figure 1:**
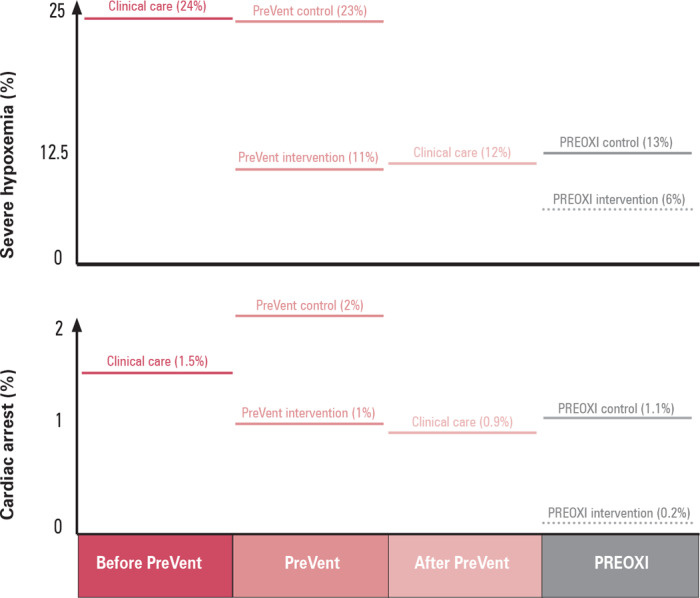
Learning Healthcare System Approach to Tracheal Intubation

A total of 1,301 patients were enrolled at 24 sites over 19 months. The results of the trial, published in the *New England Journal of Medicine* in June 2024, found that preoxygenation with noninvasive ventilation reduced the risk of hypoxemia during tracheal intubation by half, compared to preoxygenation with an oxygen mask.^33^ By preventing hypoxemia, preoxygenation with noninvasive ventilation also appeared to prevent cardiac arrest, the most serious complication of emergency tracheal intubation (see figure 1). By August of 2024, the results of the trial had been incorporated into expert recommendations and clinician references such as UpToDate.^34^

### The RSI trial

The Randomized Trial of Sedative Choice for Intubation (RSI) is an ongoing 2,364‐patient trial comparing ketamine and etomidate for induction of anesthesia during emergency tracheal intubation. Both of these drugs are FDA approved for induction of anesthesia during emergency tracheal intubation and are commonly used in clinical care.^35^ The trial protocol was completed in July 2018. Because the trial interventions are FDA‐regulated medications, the investigators consulted with the FDA about whether the trial could be conducted with a waiver of informed consent. While FDA regulations did not include an option to waive informed consent for minimal risk research until December 2023, in July 2017, the FDA issued guidance stating they would not object to the use of a waiver of informed consent if an IRB determined that the OHRP criteria to waive consent for minimal risk research were satisfied. In this case, however, the FDA recommended that the trial be conducted under EFIC instead of a waiver of informed consent.

To pay for the additional expenses of conducting the trial under EFIC instead of a waiver of informed consent, the investigators obtained two federal grants—one for approximately $1 million in August 2020 for the initial enrolling site and one for approximately $8 million in November 2023 to complete the trial at five additional health systems. The trial protocol and planned processes for community consultation and public disclosure were submitted to the IRB in March 2021. The IRB deferred review until approval of the IND application by the FDA, which occurred in June 2021. The IRB approved initiation of community consultation and public disclosure activities at the initial site in October 2021. Results of community consultation and public disclosure processes at the initial site were approved by the IRB in February 2022, and the first patient was enrolled in April 2022. The community consultation and public disclosure processes were initiated for the additional five sites that planned to participate in the trial immediately after funding was obtained in November 2023 and were completed in August 2024.

Because of the costs and infrastructure required to execute pretrial EFIC requirements at each site and the requirement for a research infrastructure capable of in‐person notification of patients being enrolled 24 hours per day, seven days per week, the six sites participating in the trial are large, academic medical centers with large research teams. All of the smaller, community‐based and rural sites within the Pragmatic Critical Care Research Group are not participating in the trial. As of October 2024, the six planned sites were actively enrolling patients. As in the PREOXI trial, patients enrolled in the RSI trial or their legally authorized representatives receive an IRB‐approved document that describes the patient's participation in the trial, the rationale for the trial, the two treatment approaches being compared and their risks and benefits, and other aspects of the research. As in the PREOXI trial, the document provides the contact information for study personnel and an opportunity to discontinue participation. Unlike the PREOXI trial, and to comply with EFIC regulations, notification forms in the RSI are delivered by research personnel, rather than by treating clinicians. As of October 1, 2024 (more than six years after completion of the initial trial protocol), the RSI trial has enrolled less than half of the planned sample size.

The PREOXI trial and the RSI trials—which were conducted at the same time, by the same research network, at the same sites, studying the same clinical procedure—provide a dramatic illustration of how the regulatory approach affects the total cost and duration of comparative effectiveness trials. Obtaining informed consent from participants was not feasible in either trial. Both trials provided information to patients and families about trial participation after enrollment using IRB‐approved notification forms. The rate at which patients and families declined participation or withdrew from each study were exceedingly low (see table 1). However, the PREOXI trial, which was conducted with a waiver of informed consent, was completed in two years while the RSI trial will ultimately require more than eight years to complete. The cost of pretrial EFIC procedures, increased regulatory burden during trial execution, and the requirement that all participants be notified by research personnel, rather than clinical personnel, resulted in the exclusion of smaller, community‐based and rural sites from the RSI trial and increased total cost to nearly four times as much per participant as the PREOXI trial.

**Table 1:** Comparison of Airway Trials Conducted under EFIC and Waiver of Informed Consent

*Trial*	*PREOXI*	*RSI*
Consent mechanism	Waiver of informed consent	Exception from informed consent (EFIC)
Sample size	1,300 patients	2,364 patients
Sites	24	6
Protocol completed	September 2021	July 2018
First patient enrolled	March 2022	April 2022
Patient notification	IRB‐approved patient and family notification sheet	IRB‐approved patient and family notification sheet
Number of patients who declined participation or withdrew	2 (0.2%)	4 (0.4%)*
Final patient enrolled	October 2023	Est: December 2026
Trial duration	2 years	>8 years
Approximate cost	$1.5 million (approximately $1,000/patient)	$9 million (approximately $4,000/patient)
Results	Preoxygenation with noninvasive ventilation reduces hypoxemia and cardiac arrest	Unknown—awaiting trial completion

* As of October 1, 2024, when enrollment was approximately 50% complete.

The time required to complete these trials is also important because patients are receiving each of the treatments being compared in current clinical care every day. The PREOXI trial's finding that the use of noninvasive ventilation decreased the incidence of cardiac arrest by nearly 1% means that, among the approximately five million patients who undergo emergency tracheal intubation each year in the US, using noninvasive ventilation for preoxygenation could prevent up to 40,000 cardiac arrests each year. Had the PREOXI trial been conducted under EFIC rather than waiver of informed consent and experienced a similar 6‐year delay to the RSI trial, nearly a quarter of a million patients would have experienced a cardiac arrest during intubation in clinical care that could potentially have been prevented. Moreover, the costs and burdens of EFIC may have prevented the PREOXI trial from ever being conducted at all—such that 40,000 patients would experience potentially preventable cardiac arrests each year indefinitely due to challenges meeting the requirements that are included in the EFIC regulations to protect research participants from risk.

### Effect of regulatory pathway on trial conduct and impact on clinical care

By conducting a series of trials in emergency tracheal intubation and implementing the treatments found to be effective into clinical care as part of subsequent trials, the Pragmatic Critical Care Research Group has applied a Learning Healthcare System framework to emergency care research.^1^ When the Preventing Hypoxemia with Manual Ventilation during Endotracheal Intubation (PreVent) trial demonstrated in 2019 that positive pressure ventilation after induction reduced the incidence of hypoxemia during intubation,^36^ it was added as a standard intervention in the next trial conducted by the network.^37^ Figure 1 shows how the rates of hypoxemia and cardiac arrest have decreased in the medical intensive care unit at the network's Coordinating Center through the conduct of the PreVent and PREOXI trials and the application of their results to clinical care. Systematically conducting rapid, efficient, comparative effectiveness trials under a waiver of informed consent and implementing the results into clinical care in the intensive care unit has reduced the incidence of severe hypoxemia during intubation by 75% and reduced the incidence of cardiac arrest during intubation by nearly 90%. Results like these show the harm to which patients are systematically and unknowingly exposed as a part of clinical care and demonstrate that structuring the arbitrary variation that occurs in clinical care into random variation in comparative effectiveness trials can protect future patients from these harms.

## DISCUSSION AT THE NIH WORKSHOP

The workshop included significant discussion regarding which regulatory approaches to comparative effectiveness trials in the emergency care setting were permitted under the current Common Rule and FDA regulations. Attendees agreed that the current regulations for clinical research were written with a view toward drug and device discovery, rather than for large‐scale trials embedded within clinical care comparing commonly used treatments or strategies among thousands, tens of thousands, or even hundreds of thousands of patients. There was broad agreement that such comparative effectiveness trials, while offering the potential for substantial societal benefit, reveal a gap in the current regulations.

When discussing the application of EFIC to comparative effectiveness trials, attendees noted that guidance from the FDA on EFIC can be interpreted as recommending that the extent of community consultation and public disclosure be matched to the risks of trial participation, suggesting that a more efficient pretrial process may be permissible for low‐risk comparative effectiveness trials under current regulations. Three barriers were identified, however, when considering the use of EFIC for comparative effectiveness trials: (1) the requirement for IND or IDE for FDA‐regulated interventions; (2) the need for a robust research infrastructure to meet the requirements that patients be provided with an opportunity to decline participation before enrollment and be notified by research staff following enrollment; and (3) the narrow focus of EFIC on life‐threatening conditions. With respect to this latter criterion, many important areas of uncertainty in current emergency care involve conditions in which obtaining informed consent is infeasible, but the condition is not immediately life‐threatening. For example, the Prehospital Analgesia INtervention (PAIN) Trial is a multicenter trial being conducted by the Linking Investigations in Trauma and Emergency Services network. At the conference, PAIN investigators shared that the trial had been initially designed to compare ketamine and fentanyl for prehospital treatment among a broad population of patients with pain following trauma. In consultation with the FDA during the IND process, the FDA raised concerns that pain was not a life‐threatening condition, and the investigators modified the inclusion criteria to adhere to the current EFIC guidance. Ultimately, the trial was approved after limiting recruitment to patients with traumatic pain and compensated shock, changing the scientific question and excluding most patients who receive prehospital treatment for pain. In the discussion of this case, there was reluctance from many attendees to expand the scope of EFIC beyond emergency research of an immediately life‐threatening condition.

To compare existing therapies, the majority view was that a better path may be defining conditions under which comparative effectiveness research may be conducted under a waiver or alteration of informed consent. It was noted that a potential opportunity for such a clarification was imminent as the FDA is currently preparing additional guidance “to assist IRBs in applying the criteria for waiver or alteration of informed consent requirements.”^14^ Subsequent conversations focused on what criteria future regulations might use to determine the type of research that could be conducted with a waiver of informed consent. Potential criteria proposed at the NIH workshop are provided in table 2.

**Table 2:** Proposed Criteria for Evaluating Whether a Comparative Effectiveness Trial Is Minimal Risk

*Domain*	*Criteria*	*Relevant criteria in an example trial comparing two common IV fluids*
Treatments	The study compares interventions (i) to which patients would likely be exposed in clinical care even if not participating in research and (ii) the receipt of which varies by arbitrary factors in clinical care.	The two intravenous fluids being compared are commonly used in clinical care and the choice between the two varies based on the specialty of the clinician, hospital formulary, and geographic region rather than patient characteristics.
Patient autonomy	The study compares interventions (i) between which a reasonable patient would not be anticipated to have a meaningful preference and (ii) on which patient input is not ordinarily sought in clinical care.	Patients’ experiences would be identical with receipt of each fluid, patients do not provide input into the choice between the two fluids in clinical care, and patients are frequently unaware even that two different types of fluid exist.
Nontreatment study procedures	Any study procedures beyond the interventions being compared present no more than minimal risk.	The study uses only data collected in the electronic health record as part of clinical care and involves no additional tests such as blood collection, X‐rays, or other interaction with research staff.
Clinician autonomy	For interventions between which a clinician would choose in clinical care, clinicians retain autonomy during the research to overrule group assignment to administer to the patient any treatment that they believe is optimal for the patient at any time.	For a patient participating in the trial, the clinician may elect to use the nonassigned fluid at any time if determined to represent optimal care for a given patient.

## SUMMARY

We believe that the inability to efficiently conduct comparative effectiveness trials leads to arbitrary variation in clinical care that systematically exposes patients to suboptimal or harmful therapies (like the preventable cardiac arrests that were occurring unnoticed in clinical care prior to the PREOXI trial). Generating the comparative effectiveness data needed to improve clinical care will require revising ethical and regulatory oversight practices and related guidance so as to allow this variation to be structured through comparative effectiveness trials generating knowledge, reducing variation, and improving outcomes over time. The majority of attendees at the NIH workshop perceived that the current status quo of arbitrary variation is ethically problematic, that comparative effectiveness trials addressing arbitrary variation generate socially valuable knowledge, and that at least some comparative effectiveness trials could appropriately be conducted under a waiver of informed consent. Attendees uniformly agreed that additional guidance from federal agencies is necessary and will be welcomed by the research and scientific community to better understand what type of comparative effectiveness research may be conducted under the minimal risk waiver or alteration of informed consent, following the publication of the FDA's final rule, “Institutional Review Board Waiver or Alteration of Informed Consent for Minimal Risk Clinical Investigations.”^14^

## DISCLOSURES

Jonathan D. Casey was supported in part by grants from the National Center for Advancing Translational Sciences of the National Institutes of Health (NIH) (U24TR004437‐02) for the Vanderbilt Trial Innovation Center and grants from the National Heart, Lung, and Blood Institute of the NIH, the Department of Defense, the Patient‐Centered Outcomes Research Institute, and the Greenwall Foundation. Matthew W. Semler was supported in part by a grant from the Greenwall Foundation. Stephanie R. Morain was supported in part by a grant from the Greenwall Foundation.
